# Regional Left Ventricular Fiber Stress Analysis for Cardiac Resynchronization Therapy Response

**DOI:** 10.1007/s10439-022-03030-y

**Published:** 2022-07-27

**Authors:** Mohammad Albatat, Henrik Nicolay Finsberg, Hermenegild Arevalo, Joakim Sundnes, Jacob Bergsland, Ilangko Balasingham, Hans Henrik Odland

**Affiliations:** 1 Intervention Centre, Oslo University Hospital, Oslo, Norway; 2 Institute of Clinical Medicine, University of Oslo, Oslo, Norway; 3 Department of Computational Physiology, Simula Research Laboratory, Fornebu, Norway; 4 Department of Electronic Systems, Norwegian University of Science and Technology, Trondheim, Norway; 5 Department of Cardiology and Department of Pediatric Cardiology, Oslo University Hospital, Oslo, Norway

**Keywords:** Cardiac resynchronization therapy, Computational cardiology, Electrophysiology heart failure

## Abstract

Cardiac resynchronization therapy (CRT) is an effective treatment for a subgroup of heart failure (HF) patients, but more than 30% of those selected do not improve after CRT implantation. Imperfect pre-procedural criteria for patient selection and optimization are the main causes of the high non-response rate. In this study, we evaluated a novel measure for assessing CRT response. We used a computational modeling framework to calculate the regional stress of the left ventricular wall of seven CRT patients and seven healthy controls. The standard deviation of regional wall stress at the time of mitral valve closure (SD_MVC) was used to quantify dyssynchrony and compared between patients and controls and among the patients. The results show that SD_MVC is significantly lower in controls than patients and correlates with long-term response in patients, based on end-diastolic volume reduction. In contrast to our initial hypothesis, patients with lower SD_MVC respond better to therapy. The patient with the highest SD_MVC was the only non-responder in the patient cohort. The distribution of fiber stress at the beginning of the isovolumetric phase seems to correlate with the degree of response and the use of this measurement could potentially improve selection criteria for CRT implantation. Further studies with a larger cohort of patients are needed to validate these results.

## Introduction

HF is a major health problem affecting 26 million people and increases in prevalence with age.^34^ About one-third of HF patients have ventricular conduction disorders causing dyssynchronous ventricular contraction and relaxation patterns.^24^ CRT may improve pumping mechanisms, HF symptoms, quality of life, and reduce mortality.^10^ CRT utilizes an implantable electrical pacemaker that paces both ventricles simultaneously through pacing leads in the right ventricle and a coronary sinus branch (CS) located on the left ventricular surface. An important problem with CRT is that 30% of patients with a class Ia indication according to guidelines, do not benefit from the therapy (non-responders). This is thought to be due to several factors, including an improper selection of patients and suboptimal activation of the left ventricle (LV).^20^ Around 10% of patients get worse after CRT implantation.^9^

Non-responders are defined as patients who do not show evidence of reverse remodeling of the LV, with less than 15% decrease in end-systolic volume (ESV) at 6 months after CRT implantation, as defined by Refs. 9 and 29. The high rate of non-responders for CRT results in high costs to payors and potential adverse effects to patients and therefore contribute to a reluctance to utilize this therapy which is effective in two-thirds of the patients. There is a need for a better understanding of the underlying mechanism of dyssynchronous HF and to identify reliable pre-procedural predictors for CRT efficacy to (1) avoid implantation in non-responders; (2) increase utilization of CRT appropriately; (3) improve CRT efficiency in patients that get implants.

The guidelines for the selection of patients for CRT are based on large randomized trials which included a broad spectrum of patients.^30^ These guidelines have limitations demonstrated by the high number of non-responders. Defining parameters that can reliably predict the outcome of CRT and determine optimal electrode placement have so far had limited success.^11^ Echocardiographic measures of dyssynchrony have not been reliable in predicting CRT response.^9^ Assessment of regional mechanical function to predict CRT response seems promising.^18,31,35,38^ Studies have shown that dyssynchronous ventricles disperse myocardial work and that modification of myocardial workload is a key factor in the reverse remodeling seen after CRT.^13,14,36^ One study suggests that the so-called “wasted work”-parameter during the isovolumetric phase of the heart cycle may be a predictor.^1,37^ Wasted work is the percentage of the contractile segments that perform negative work (lengthening), during systole compared to the regions performing positive work (shortening). Calculating wasted work requires complex measurements, including invasive pressure and volume. To obtain it non-invasively, regional work is estimated by multiplying segmental strain (rate of segmental shortening using echocardiography) with arterial pressure, which is assumed to be equal to the left ventricular pressure at each sampling time. This is a considerable limitation because accurately calculating regional work requires the integration of wall strain and wall stress. Stress is particularly important in this application as it incorporates regional wall thickness and curvature.

Advances in computational medicine and detailed heart modeling^26^ give us the potential to noninvasively measure regional wall stress and analyze the effect of CRT in silico, and compare results to clinical outcomes. In this paper, we propose a new patient-specific computational model to compute LV regional stress at the beginning of the isovolumetric phase and investigate its predictive value for the success of CRT response, using retrospective patient data.

The isovolumetric phase is the period between the mitral valve closure (MVC) and aortic valve opening (AVO), in which the LV volume remains constant while the LV pressure increases from stress generated by the LV wall. Ideally, the wall stress at the beginning of the isovolumetric phase is *uniformly distributed* both in time and space for optimal LV contraction without wasted work. However, with left bundle branch block (LBBB), the activation of the myocardium during the isovolumetric phase is dispersed with regional delays allowing contraction with shortening of myofibrils before full activation of the myocardium occurs. We hypothesize that higher variance in regional stress during the isovolumetric phase, implies greater resynchronization potential, i.e., if the LV wall stress is distributed non-uniformly at the beginning of the isovolumetric phase, there would be greater potential for success of CRT, which may redistribute the stress and achieve uniform contraction. We aim to elucidate to what extent the distribution of regional stress varies between controls and LBBB patients, and between responders and non-responders among CRT patients.

## Methods

The computational model used in this study is described in Ref. 16 and briefly summarized here. 4D echocardiography was obtained from seven LBBB patients with indication for CRT who had been enrolled in the Impact Study^22^ and seven healthy volunteers. The study received ethical approval and consent was obtained from every patient. The software package EchoPac (GE healthcare, MA, USA) was used to calculate LV volume and strain in the longitudinal, radial, and circumferential direction for each of the 17 segments of the LV defined by the American Heart Association (AHA).^8,32^ LV pressures of the patients were measured invasively during CRT- implantation, while the pressure for the healthy subjects was set using reported values of healthy LVs synchronized to valve events for each control. Echocardiographic images were used to create 3D tetrahedral meshes of the LV, and rule-based fiber orientation was assigned using the histologically validated algorithm described in Ref. 6. All patients received CRT in the form of biventricular pacing. The end-systolic volumes (ESV) of the seven patients were obtained 6 months after implantation. To assess CRT response, the percentage ESV decrease between pre-implantation and 6 months post-implantation was calculated:$\Delta \mathrm{ESV}=({\mathrm{ESV}}_{\mathrm{pre}}-{\mathrm{ESV}}_{\mathrm{post}})/{\mathrm{ESV}}_{\mathrm{pre}}.$

**Figure 1:**
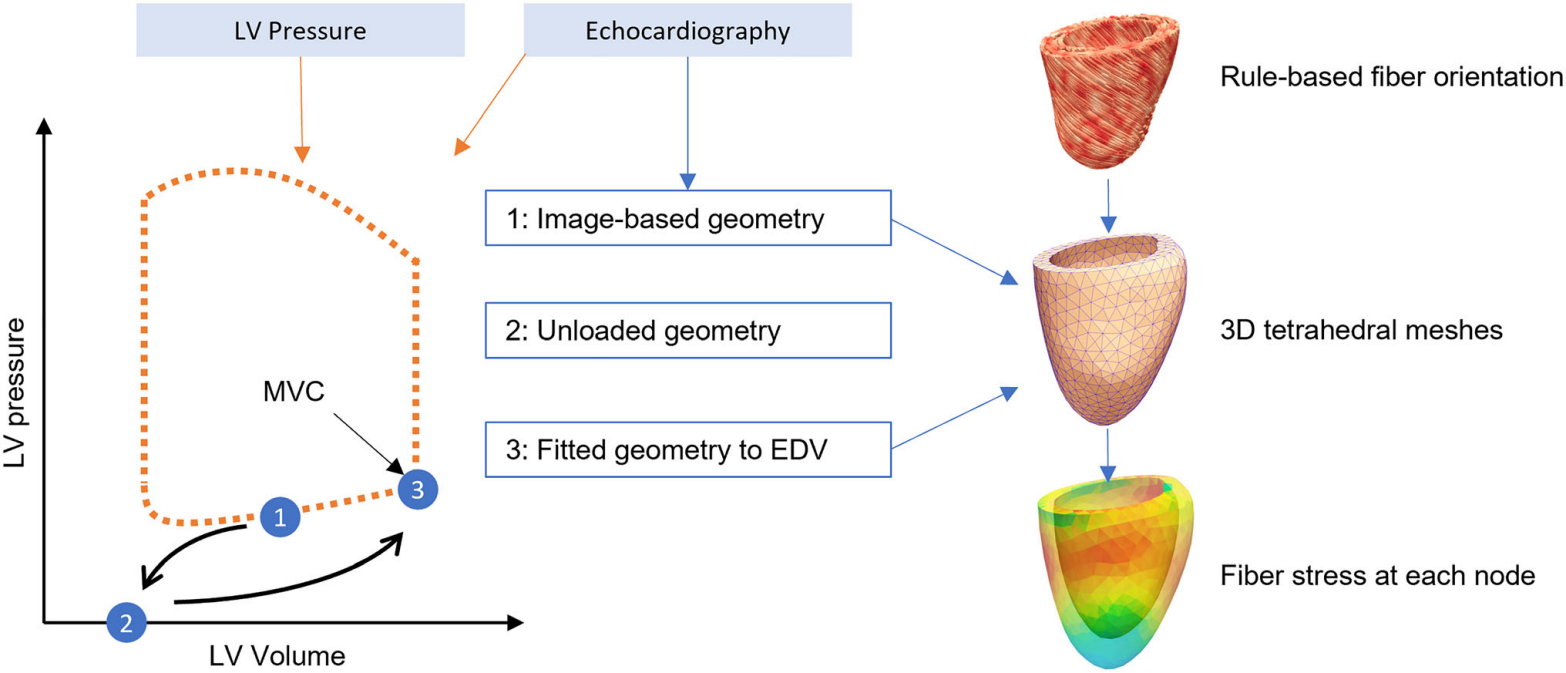
The model creation process. LV pressures, volumes, and strains were obtained for each subject at multiple time points throughout a cardiac cycle, as illustrated by the pressure–volume graph. (1) 4D echography image from the heart at atrial systole was used to create a 3d tetrahedral model of the LV and fiber directions were assigned from rule-based fiber orientation. (2) The image-based model was unloaded using the backward displacement method^7^ until zero pressure was reached. (3) The measured volumes, pressures, and strains were used to estimate the material properties of the LV and inflate the unloaded geometry until reaching the end-diastolic pressure. The stress at each node of the simulated model was then calculated, as shown on the model in the bottom right corner, where the color represents the stress at each node.

The timepoint corresponding to the start of atrial systole was used for the initial geometry of the model. Then the unloaded geometry of the LV, defined as the geometry when no force is exerted on the LV, was estimated using the backward displacement method.^7^ The measured data (volume, strain, and pressure) was then assimilated into active- and passive- phases.^4^ In the passive phase, no contraction was considered as the LV relaxes. The linear isotropic material parameter (as described in Ref. 16, as a parameter representing the stiffness of the extracellular matrix) of a transversally isotropic version of the incompressible, hyperelastic model described in Ref. 21 was estimated iteratively by minimizing the difference between the simulated and measured volumes at each timepoint until end-diastole was reached. The optimized passive material parameter was fixed for the whole ventricle. Figure 1 provides an overview of the model creation process.

For each of the timepoints, stress was calculated at each node in three dimensions. We analyzed the stress component along the fiber direction (fiber stress) because it is the dominant direction in the stress development of the myocytes.^5,17^ Fiber stress was calculated by first computing the Cauchy Stress tensor^21^ and extracting the component along the fiber axis. The computational model was implemented using the finite element framework FEniCS^23^ using Taylor–Hood finite elements (P2-P1) for the displacement and hydrostatic pressure.

The average stress along the direction of the fibers was computed by integrating over the domain and dividing by the volume of the domain. To quantify spatial stress distribution, the SD of the stress applied to all the mesh nodes was calculated at MVC (SD_MVC) for all the 14 subjects.

A low SD value indicates that the stress values are concentrated around the mean, while a higher SD value indicates that the stress values are spread over a wider range. MVC was chosen since it is the start point of the isovolumetric phase and is clearly defined in the pressure–volume loops (see Fig. 1). During the isovolumetric phase after the MVC, although the volume is constant, some segments will start to contract, and others stretch and naturally cause an increasing stress variance towards systole, reflected by a higher SD. Any stress variance at the beginning of the isovolumetric phase is caused by underlying dyssynchrony that is transmitted to the systole.

## Results

**Figure 2:**
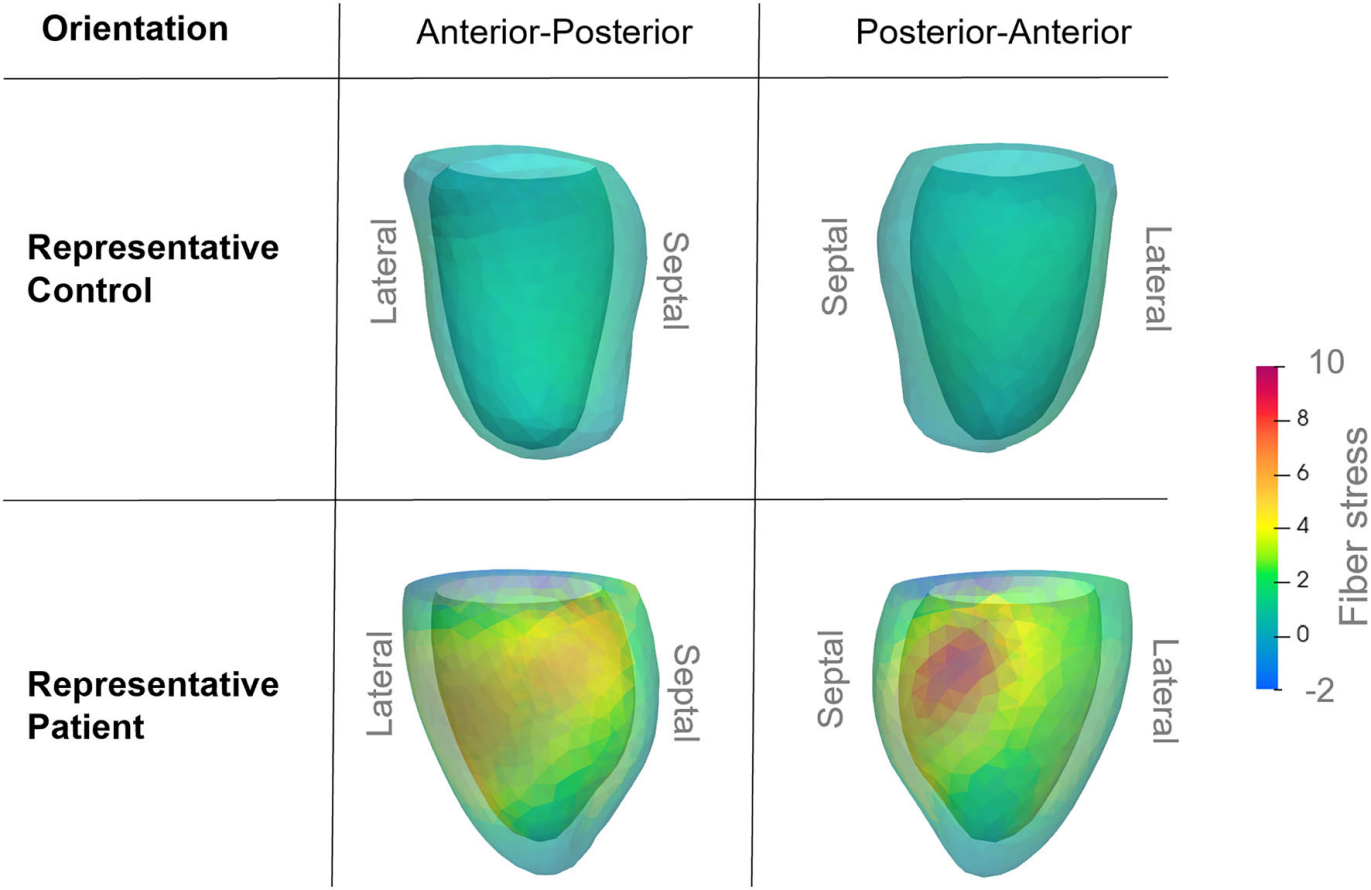
Simulation results of a representative patient and control displayed from two angles. The color represents the calculated fiber stress at each node at MVC. The more varied colormap of the patient signifies a higher variance in stress distribution

To visualize the fiber stress distribution, the nodes of the geometric model were color-coded based on their fiber stress at MVC, as seen in Fig. 2, where a model of a representative patient and control is shown. The controls had uniform color distribution, while patients had uneven distribution, signifying higher variance in the fiber stress.

**Figure 3:**
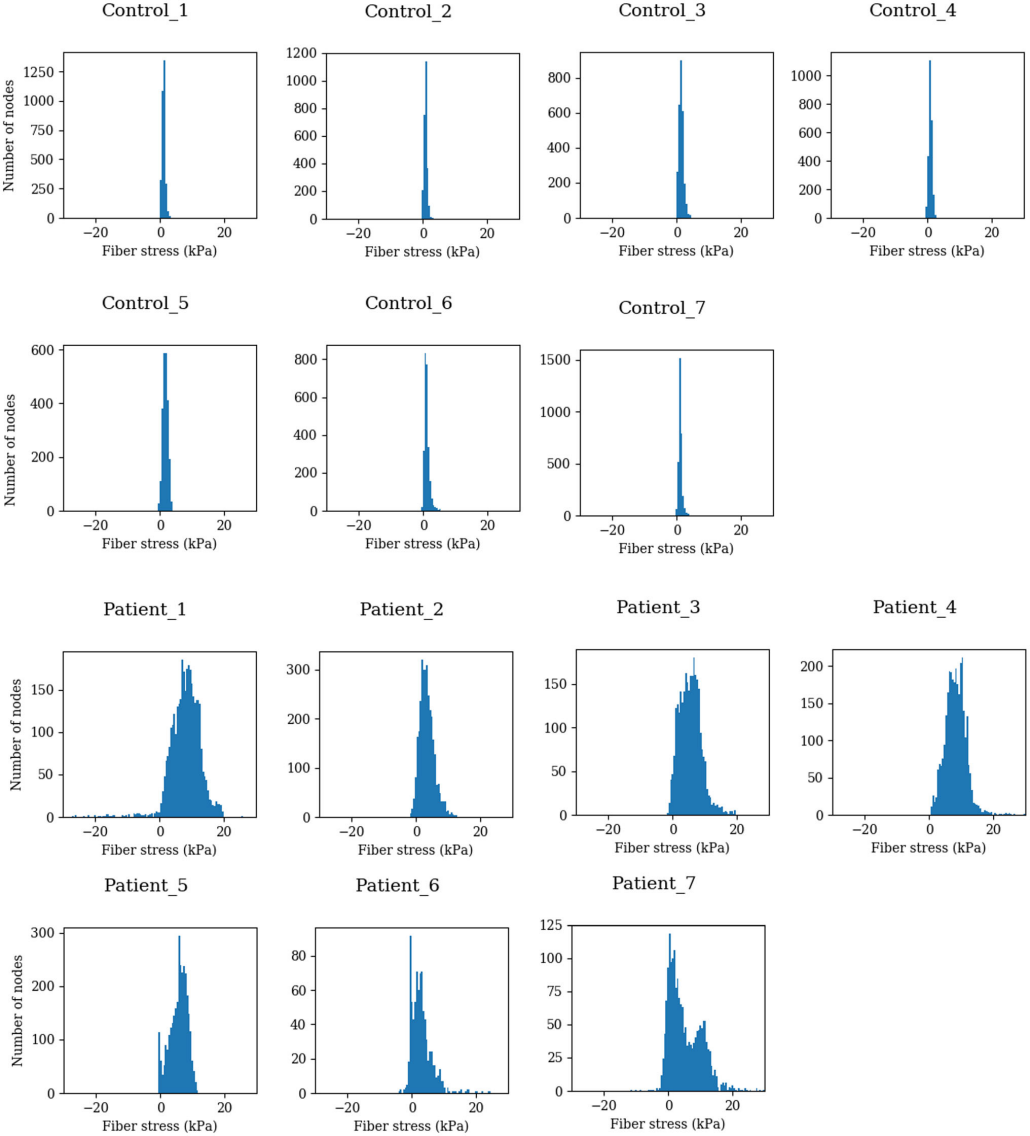
Histograms of the fiber stress at MVC. The values of the fiber stress are windowed in a 1 kPa window and plotted against the number of nodes with a stress value within the window.

Since it is hard to visualize the distribution of stress in the 3D structure, histograms of the stress distribution throughout the LV nodes were created to illustrate the distribution. As shown in Fig. 3, histograms of patients show a wider spread than in controls. This is captured quantitively by higher SDs in patients. Table 1 shows the measurements for all subjects. SD_MVC is significantly higher in patients compared to control, *p* < 0.001 (by one-way ANOVA using SPSS Statistics software. This means SD_MVC can clearly distinguish between the LBBB patients and the controls.

**Table 1:** Results summary.

Controls		Patients					
Subject	SD_MVC	Subject	Ischemic	SD_MVC	ESV_pre	ESV_post	ΔESV (%)
Control1	0.43	P1	Yes	4.45	229	144	**37.12**
Control2	0.88	P2	Yes	2.08	106	33	**68.87**
Control3	0.62	P3	Yes	2.88	87	50	**42.53**
Control4	0.44	P4	Yes	2.71	254	122	**51.97**
Control5	1.28	P5	Yes	2.53	138	50	**63.77**
Control6	0.68	P6	No	3.28	87	73	**16.09**
Control7	0.45	P7	No	5.2	363	413	*-13.77*

The table shows the SD_MVC for each subject and the pre-implantation (ESV_pre) and 6-months post-implantation (ESV_post) ESV for the patients. ΔESV is the percentage reduction in ESV. Note that SD_MVC of the patients is obtained pre-implantation

Bold values indicate responders, while italic value is the non-responder

The degree of response to CRT is variable between patients. The ΔESV and its relation to SD_MVC is therefore of interest. Using Pearson Correlation, we demonstrated significant correlation between ΔESV and SD_MVC with *r* = − 0.859, *p* = 0.018. This means that the higher the SD_MVC is, the lower the response is. As seen in Table 1, the two patients with the lowest response (Lowest ΔESV) were the only ones that were ischemic.

**Figure 4:**
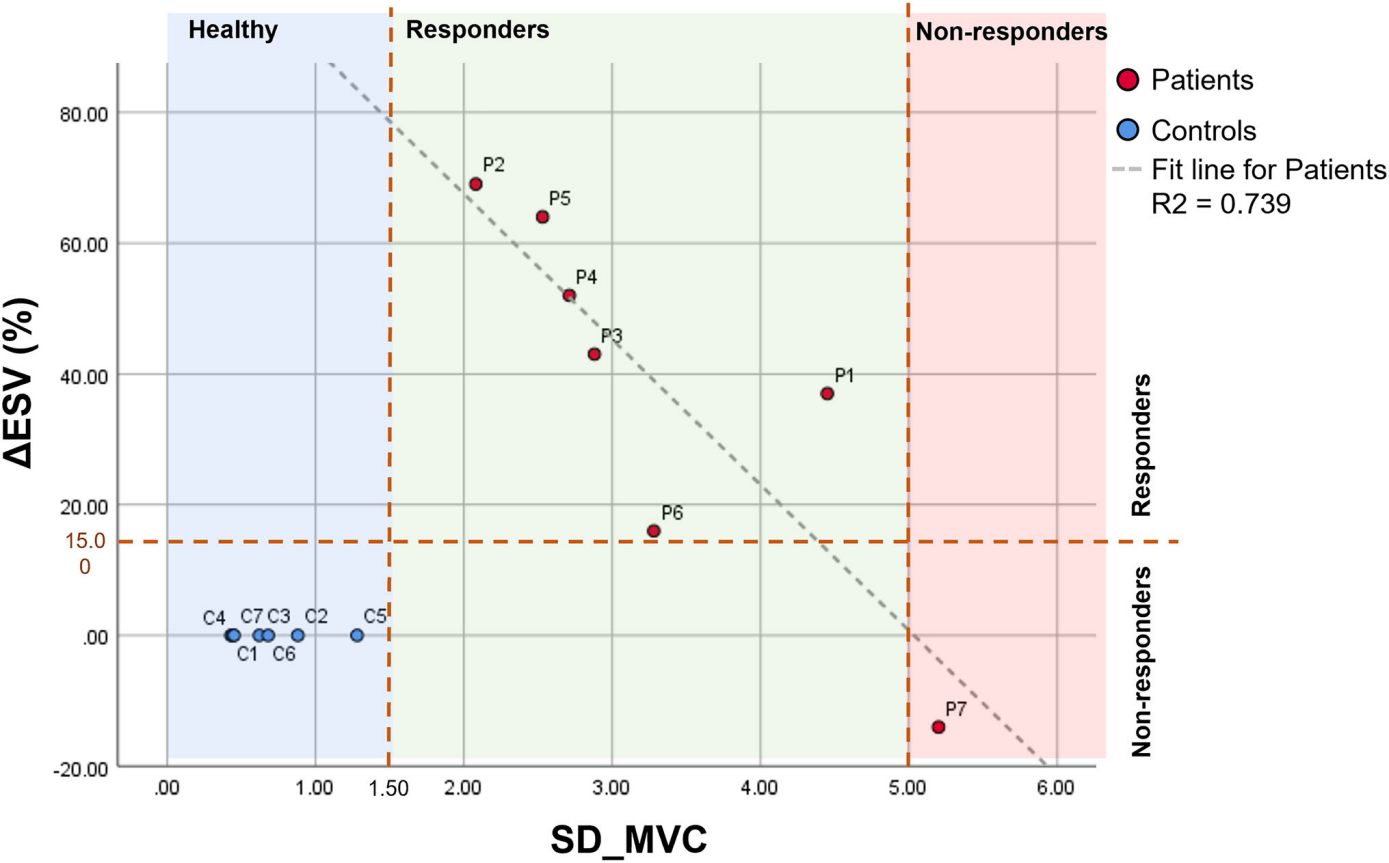
Response rate to CRT (% improvement in ESV = ΔESV) against SD_MVC with linear regression fit line. Responders are defined with ΔESV above 15% (horizontal dotted line). All responders had SD_MVC between 1.5 and 5 (vertical dotted lines). Higher SD_MVC values were associated with lower ΔESV. The controls are included in the graph with ΔESV = 0 to show them in context.

Figure 4 shows the correlation between ΔESV and SD_MVC graphically. Lower SD_MVC is associated with higher ESV reduction i.e., better response. If we define response to CRT as being ΔESV > 15%, Patient 7 is considered a non-responder. Drawing lines on the graph at SD_MVC = 1.5 and 5 separates the control and the non-responder as seen in Fig. 4. All the controls had SD_MVC below 1.5, while all responders had SD_MVC between 1.5 and 5 with the highest responders (highest ΔESV) having the lowest SD_MVC and the lowest responders having the highest SD_MVC. The single non-responder had an SD_MVC of more than 5.

## Discussion

Responders to CRT experience decreased mortality, improvement in symptoms, quality of life, and echo-cardiography function 6 months after implantation.^25^ Non-responders get no effect from this costly and invasive therapy and are exposed to potential adverse effects and delay of alternative treatment regimens. Since CRT is used mainly in patients with HF, unsuccessful CRT may delay the initiation of alternative therapy, which could include implantation of a left ventricular assist device, or heart transplantation.

The parameter, SD_MVC, described in this paper, demonstrates that there are considerable differences in stress distribution shortly after ventricular activation occurring at MVC in patients with LBBB compared to controls. This demonstrates that myocardial function is impacted by dyssynchrony already during the earliest phase of systole. Reducing dyssynchrony with CRT can improve systolic function by increasing the uniformity of the stress distribution throughout the LV. Reducing dyssynchrony already at MVC is, therefore, an important aim of CRT.^12^

LBBB results in high SD_MVC in our small number of patients. The single non-responder had the highest SD_MVC while patients with lower SD_MVC responded better as shown in Fig. 3. The reason for this is unclear as we initially hypothesized that a large SD_MVC and corresponding dyssynchrony would respond better. The higher values of SD_MVC could indicate the myocardial dysfunction is not associated with electrical activation but with other comorbidities such as aneurysms or scarred regions. The non-responder in our small series had the highest SD_MVC possibly due to ischemic myocardial disease not responsive to CRT. This could potentially be due to non-recruitable, passive myocardial tissue with mechanical dyssynchrony. That patient (P7 in Fig. 3) had the highest ESV of all patients both before and after implantation.

The width of the QRS is a valuable measure for differentiating LBBB patients from healthy individuals but is a poor predictor of response to CRT.^28^ Additional selection criteria are needed, and our study indicates that measuring the SD_MVC may predict CRT outcomes. The early phase of contraction is less dependent on afterload, and wall stress during this phase of contraction could therefore to a larger extent reflect the direct effects of electrical activation. Hence, more interest should be put into the assessment of this early phase to understand dyssynchronous heart failure.^27^ While SD_MVC may have a value as a predictor of response, individualized treatment is a key determinant factor. The CRT outcome is largely dependent on the positioning of the pacing electrodes.^2,3^ Using repeated measurements of SD_MVC may be important to define an optimal pacing site. Reducing SD_MVC is likely to lead to the reversal of the pathogenic mechanisms thereby causing reverse remodeling and potentially improving the clinical syndrome of heart failure.

Presently, the simulations necessary to obtain the required modeling on a standard desktop computer take several hours. The use of 4D echocardiography in conjunction with improvements in algorithms and the evolution in performance of standard computers may reduce computation times, SD_MVC could then be calculated to support lead placement during ongoing interventions.

The challenges in computational physiology include the validation of data obtained from models. The novelty of the fiber stress measure and the fact that it is currently impossible to measure it in vivo makes it difficult to verify the direct clinical impact of the simulations. This has a low impact on our analysis since the distribution of the measure and not absolute values were used. The simulation algorithm is constructed to assimilate clinical data thereby validating the model to an extent. The strong correlation between the simulated volumes and the measured volumes demonstrated in Fig. 3 in Ref. 16, encourages us to pursue further clinical studies.

This study has demonstrated that measurements obtained during the earliest phase of systole may carry prognostic information about CRT results. Measurement of early systolic markers, before aortic valve opening, are less dependent on afterload and hence less influenced by confounding factors. Markers of mechanical function, like wall stress or work, may provide value as response markers as well for the demonstration of reversible disease with the potential to determine long-term outcomes. Accurate determination of long-term outcomes may help improve the selection of patients and reduce the socioeconomic burden of non-response to treatment.

The simulations performed in this study required invasive pressure measurements in addition to noninvasive imaging. The use of non-invasive pressure assessment, described in Ref. 33, may improve workflow and make use of simulations a preoperative selection tool.

## Limitations

Although our results show promise, the sample size is small, and larger clinical investigations are required before our method could be used as a clinical tool.

Another limitation is the low sampling frequency of pressure and strain values, which were recorded approximately every 30 ms. This relatively long time interval may have led to the loss of valuable information between timepoints. Echocardiography was recorded separately from pressures and synchronized afterward using well-defined valvular events. Preferentially, measurements should be obtained simultaneously with a higher sampling frequency.

The response to therapy was assessed using the reduction in ESV 6-months after implantation and we do not have data on post-implant measurements of SD_MVC. Unfortunately, the measurements required for such calculation were not part of the clinical follow-up procedure.

While ESV reduction is commonly used for the assessment of CRT outcomes, other factors often contribute to the outcome, including baseline ESV, wall thickness, myocardial scars, and other co-morbidities. The complexity of this issue is a major driver, leading both clinicians and researchers to highlight the importance of the personalization of CRT.^15,19^

## Conclusion

We have demonstrated that SD_MVC correlates with CRT response. In contrast to our initial hypothesis, high SD_MVC values are associated with a lower degree of response rate while low SD_MVC values are associated with high response rates. Despite the low patient volume, our study demonstrates that this measure has the potential to become an important clinical parameter for the selection of patients for CRT and help optimize the therapy.
